# Expression analyses of nuclear receptor genes in breast cancer cell lines exposed to soy phytoestrogens after *BRCA2 *knockdown by TaqMan Low-Density Array (TLDA)

**DOI:** 10.1186/1750-2187-4-3

**Published:** 2009-05-14

**Authors:** Samir Satih, Hélène Savinel, Nadège Rabiau, Luc Fontana, Yves-Jean Bignon, Dominique J Bernard-Gallon

**Affiliations:** 1 Centre Jean Perrin, Département d'Oncogénétique, CBRV, 28 Place Henri Dunant, 63001 Clermont-Ferrand, France; 2 Université d'Auvergne-CJP, EA 4233 Nutrition, Cancérogenèse et Thérapie anti-tumorale, 28 Place Henri Dunant, 63001 Clermont-Ferrand, France; 3 Soluscience, biopôle Clermont-Limagne, 63360 Saint Beauzire, France; 4 CHU, Service de Médecine du Travail et des Pathologies Professionnelles, 28 Place Henri Dunant, 63001 Clermont-Ferrand, France

## Abstract

**Background:**

Most of breast cancers are considered sporadic and modulation of the two major genes *BRCA1 *and *BRCA2 *expressions caused by tissue-specific somatic mutations lead to this pathology. The nutritional intake of phytoestrogens seems to reduce the risk of breast cancer and investigation of their potential as anticancer agents has increased. However, the possible mechanisms and signalling pathways of phytoestrogen action in breast cancer prevention remains unknown.

**Results:**

Using Taqman Low Density Array technology, we investigated the *BRCA2 *loss of function role in sporadic breast cancers and the links existing with soy isoflavones on a panel of nuclear receptor expression. Human breast cell lines (MCF-7, MDA-MB-231, and MCF-10a) were transfected by *BRCA2*-siRNA and treated with genistein (18.5 μM) or daidzein (78.5 μM) for 72 h. Generating the transitory knockdown of *BRCA2 *oncosuppressor, we observed different modulations in several nuclear receptor genes such as *ER*, *RAR *and *RXR*, as well as *PPAR*s and *VDR *according to the studied breast cell line. Additional isoflavone treatments showed different nuclear receptor gene modulation profiles.

**Conclusion:**

Our results seemed to implicate the oncosuppressor *BRCA2 *and the phytoestrogen pathways in different nuclear gene expressions *via *an ER-independent manner.

## Background

Breast cancer is an important public health problem worldwide. Most of breast cancers are considered sporadic, with only an estimated 5–10% due to inherited susceptibility [1]. Because *BRCA1 *and *BRCA2 *expressions are often decreased, or even absent in sporadic breast cancer, abnormal *BRCA1 *or *BRCA2 *expressions may also play a role in nonhereditary tumors [2,3]. In sporadic cases, *BRCA2 *gene is rarely inactivated, but its expression is often modulated [4]. Although *BRCA2 *function is often restricted to DNA recombination and repair, evidence is accumulating that the silencing of this gene might be of particular importance in the pathogenesis of a significant proportion of sporadic breast cancers.

Moreover, most of breast cancers are considered estrogen-dependent, and estrogens are suggested to cause breast cancer by stimulating cell growth and proliferation through receptor-mediated processes and *via *their genotoxic metabolites [5,6]. Phytoestrogens are a class of plant-derived substances that are structurally and/or functionally similar to 17β-estradiol (E_2_) [7]. Interest in phytoestrogens, more particularly soy, has been instigated by epidemiologic studies that have suggested a low incidence of breast cancer in Asian countries that have high soy intake [8,9]. It has also been suggested that soy isoflavones may influence breast cancer risk via their anti-proliferative, anti-angiogenic, anti-oxidative and anti-inflammatory properties, but the possible mechanisms of phytoestrogen actions in breast cancer prevention remained inconclusive.

Furthermore, nuclear receptors are transcriptional factors and play key roles in the regulation of gene expression and physiological activities [10]. To study this, we used RNA interference in breast human cells to down-regulate *BRCA2 *and determine its contribution to nuclear receptor expression at the transcriptional level. Two different human breast cancer cell lines (MCF-7, MDA-MB-231) and a human fibrocystic breast cell line (MCF-10a) were transfected by a pool of *BRCA2*-siRNA. They were exposed to either genistein or daidzein for 72 h. A control group of non-treated cells was included in our study. Expression pattern of 64 nuclear receptor genes were investigated using the TaqMan Low-Density Array (TLDA) technique.

## Methods

### Cell culture

MCF-7 and MDA-MB-231 breast tumor cell lines came from a pleural effusion of patients with invasive breast carcinoma [11,12]. The MCF-10a cell line was established from breast tissue of patient with fibrocystic breast disease [13]. All three human cell lines were provided by the American Type Culture Collection (ATCC). In our study, MCF-7 were cultured in RPMI 1640 media supplemented with 2 mM L-glutamine (Invitrogen), 20 μg/ml gentamycin (Panpharma), 10% fetal bovine serum (Invitrogen), 0,04 UI/ml insulin (Novo Nordisk) in a humidified atmosphere at 37°C containing 5% CO_2_. This cell line has a positive estrogen-receptor status (ERα+/ERβ+). MCF-10a cells were maintained in DMEM-F12 (Invitrogen) containing 10% horse serum (Invitrogen), 2 mM L-glutamine, 20 μg/ml gentamycin (Panpharma), 20 ng/ml epidermal growth factor (Sigma), 100 ng/ml cholera toxin (Sigma), 0,25 UI/ml insulin (Novo Nordisk) and 0,5 μg/ml hydrocortisone (Sigma) held at 37°C with 5% CO_2_. This cell line has a negative estrogen receptor status (ERα-/ERβ-). MDA-MB-231 cells were grown in Leibovitch L-15 media with 15% FBS, 20 μg/ml gentamycin (Panpharma), 2 mM L-glutamine in a 37°C humidified atmosphere without CO_2_. This cell line has a negative estrogen receptor status (ERα-/ERβ+).

### Conditional BRCA2 loss of function using small interfering RNA (siRNA)

A pool of three target-specific 20–25 nt *BRCA2*-siRNAs was chemically synthesized. The siRNA sequences used for human *BRCA2 *were 5'-CCA AGG AUG UUC UGU CAA Att-3', 5'-CAA GCU ACA UAU UGC AGA Att-3', and 5'-GAA ACG GAC UUG CUA UUU Att-3' (sc-29825, Santa Cruz Biotechnology). For siRNA treatments, 40–60% subconfluent proliferating cells were transfected with 50 nM of siRNA using the siRNA transfection reagent (sc-29528, Santa Cruz Biotechnology, California, USA). Prior studies have established that under these conditions, none of the siRNA caused cytotoxicity based on cell morphology or proliferation. To obtain mRNA *BRCA2 *maximal inhibition, we carried out 72-h incubations with *BRCA2*-siRNA and quantification was performed by real-time quantitative PCR (RT-qPCR).

### Phytoestrogen exposure

Isoflavone treatments were carried out for 72 h with 18.5 μM genistein or 78.5 μM daidzein dissolved in dimethyl sulfoxide (Sigma). These concentrations were previously obtained and corresponded to the 50% inhibition of the proliferation (IC_50_) [14]. As controls, cell lines were also conditioned in medium without siRNA transfection and without isoflavone treatments.

### RNA extraction and reverse transcription

Total RNA was isolated from MCF-7, MDA-MB-231, and MCF-10a treated cells and from control cells after 72 h. We used 1 ml RNA-PLUS (MP Biomedicals) according to the manufacturer's protocol. The RNA quality was checked by electrophoresis using a Bioanalyzer 2100 with RNA 6000 Nano LabChip^® ^and BioSizing A.02.11 software (Agilent Technologies). Five micrograms of total RNA were reverse transcribed in a total volume of 15 μl using the First-Strand DNA Synthesis Kit and performed according to the manufacturer's protocol (Amersham Biosciences). Reverse transcriptase was thermically inactivated (95°C, 10 min).

### BRCA2 knockdown analyses by quantitative real time RT-PCR

The resulting cDNA was then quantified with the TaqMan^® ^method. PCR was carried out in 96-well plates: 25 ng of cDNA and 20 μl of reaction mix containing 12.5 μl TaqMan universal PCR Master Mix (Roche) (dATP, dCTP, dGTP and dUTP, MgCl_2_, AmpliTaqGold, Amperase uracil-N-glycosylase), 200 nM of TaqMan probes corresponding to the studied gene, 400 nM of each primer and 50 nM of *18S *rRNA primers and TaqMan probe. Primers were as follows: *BRCA2*, forward: 5'-CCA AGT GGT CCA CCC CAA C-3', reverse: 5'-CAC AAT TAG GAG AAG ACA TCA GAA GC-3'; *18S*, forward: 5'-CGG CTA CCA CAT CCA AGG AA-3', reverse: 5'-GCT GGA ATT ACC GCG GCT-3' (MWG). Taqman^® ^probes were purchased from Applied Biosystems: *BRCA2*, 5'-ACT GTA CTT CAG GGC CGT ACA CTG CTC AAA-3' (FAM); *18S*, 5'-TGC TGG CAC CAG ACT TGC CCT C-3' (VIC).

Data were collected using an ABI PRISM 7700 Sequence Detector System (Applied Biosystems) for 40 cycles (95°C for 15 s, 60°C for 1 min) after an initial step of 50°C for 2 min, 95°C for 10 min. The relative amount of *BRCA2 *mRNA to *18S *rRNA was calculated as the average 2^-ΔΔCt ^where ΔC_T _= Ct_*BRCA2*_-Ct_*18S *_with data normalized to untreated controls. Two independent total RNA extractions were performed as two independent reverse transcriptions with one of the RNA extractions. All data were generated in triplicate and expressed as mean +/- SD.

### TaqMan Low Density Array (TLDA)

Predesigned TLDA called Human Nuclear Receptor Panel (64 TaqMan^® ^Gene Expression assay preconfigured in a 384-well format, Part n° 4379968 microfluidic cards, Applied Biosystems), were used in a reverse transcriptase polymerase chain reaction (RT-PCR) process using the ABI Prism 7900 HT Sequence Detection System (Applied Biosystems). The TLDA in this study was configured into 2 identical 64-gene sets in triplicate. A total of 100 μl reaction mixture with 50 μl cDNA template (100 ng) and an equal volume of TaqMan^® ^universal master mix (Applied Biosystems) was added to each line of TLDA after gentle vortex mixing. Thermal cycler conditions were as follows: 2 min at 50°C, 10 min at 94.5°C and 30 s at 97°C, and 1 min at 59.7°C for 40 cycles. The threshold cycle Ct was automatically given by SDS2.2 software package (Applied Biosystems). Relative quantities (RQ) were determined using the equation: RQ = 2^-ΔΔCt^. All data were generated two times (different TLDA plates) in triplicate and expressed as mean +/- SD.

## Results

### Down-regulated expression of *BRCA2 *by RT-PCR

**Figure 1:**
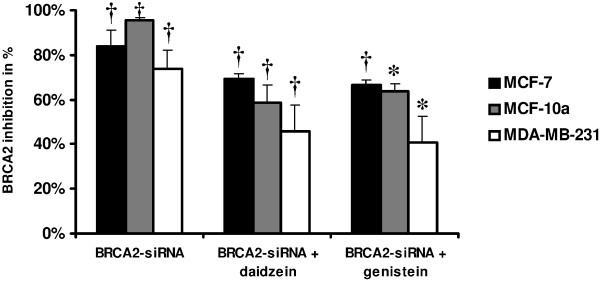
**Specific knockdown of *BRCA2 *mRNA expression measured by RT-qPCR in MCF-7, MCF-10a and MDA-MB-231 breast cell lines after *BRCA2*-siRNA, *BRCA2*-siRNA + daidzein, and *BRCA2*-siRNA + genistein 72-h treatments in comparison to controls**. The ΔΔCt method and normalization by comparison with controls were used. Values are means ± SD for n = 3 assays. Statistical analysis was carried out by Student *t*-test (*, *P *< 0.05; †, *P *< 0.01).

We down regulated the expression of *BRCA2 *by use of RNA interference, and quantification was performed by RT-qPCR in MCF 7, MCF-10a and MDA-MB-231 human breast cell lines under four conditions: 1) control, 2) siRNA-*BRCA2*, 3) siRNA-*BRCA2 *+ genistein, and 4) siRNA-*BRCA2 *+ daidzein. As shown in Figure 1, transfection of the three human breast cell lines treated with a pool of *BRCA2*-siRNA for 72 h significantly decreased the expression of *BRCA2 *mRNA compared with control cells.

### Nuclear receptor gene modulations by TLDA

Sixty-four genes (48 nuclear receptor genes and 16 endogenous controls) were successfully amplified two times in triplicate by TLDA in the four conditions described above.

**Figure 2:**
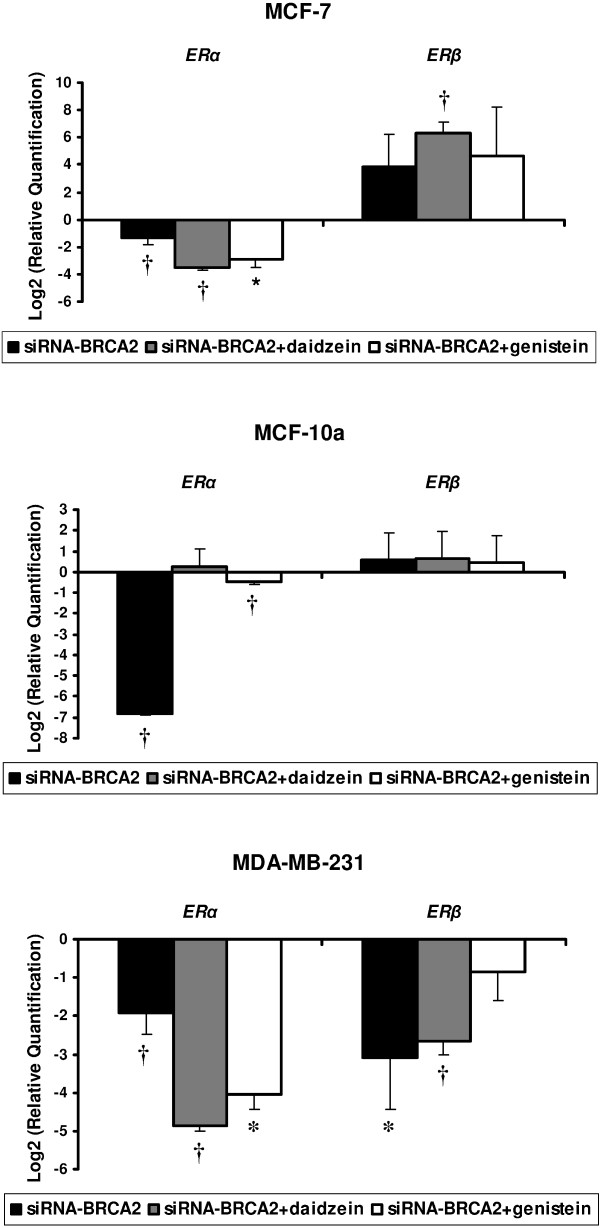
**Modulation in mRNA levels performed by TLDA for estrogen receptor genes (*ERα *and *ERβ*) with differential expression between *BRCA2*-siRNA treatment and *BRCA2*-siRNA with isoflavone exposure, compared to control cells corresponding to value 0, in MCF-7, MCF-10a and MDA-MB-231 cell lines**. Values are means ± SD for n = 3 assays. Statistical analysis were carried out by Student *t*-test (†, *P *< 0.01; *, *P *< 0.05).

Estrogen-receptor (ER) α and β expressions were analysed in the three human cell lines. In MCF-7 cell line, we observed a decreased expression of *ERα *and an over-expression of *ERβ *after *BRCA2 *knockdown, as well as after additional treatments of both genistein and daidzein. MDA-MB-231 breast cancer cells showed a decreased expression of *ERα *and *ERβ *genes in all conditions. The fibrocystic MCF-10a cell line showed a down-regulation of *ERα *expression after *BRCA2*-siRNA transfection, but no significant modulations were observed under additional isoflavone treatments. No significant modulations were observed for *ERβ *expression which is not usually expressed (Figure 2).

**Figure 3:**
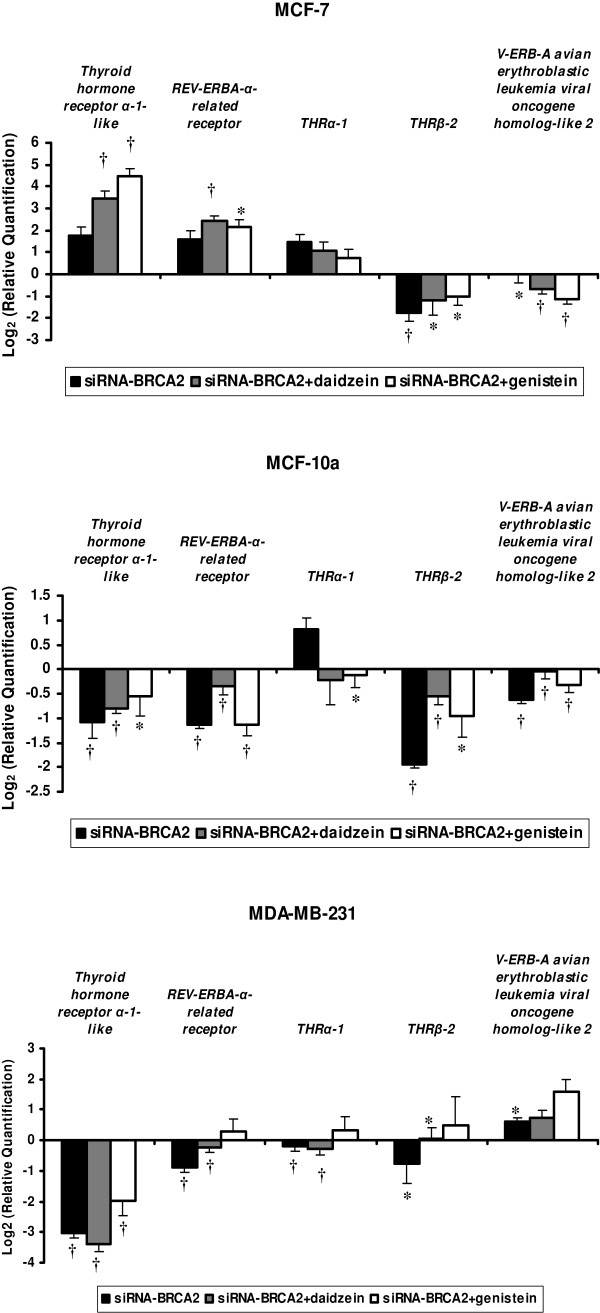
**Modulations in mRNA levels performed by TLDA for thyroid hormone receptor-related genes (*Thyroid hormone receptor α-1-like, REV-ERBA-α-related receptor, THR α-1, THR β-2, V-ERB-A avian erythroblastic leukemia viral oncogene homolog-like 2*) occurring after *BRCA2*-siRNA treatment and *BRCA2*-siRNA with isoflavone exposures, compared to control cells corresponding to value 0, in MCF-7, MCF-10a, and MDA-MB-231 cell lines**. Values are means ± SD for n = 3 assays. Statistical analysis were carried out by Student *t*-test (†, *P *< 0.01; *, *P *< 0.05).

Expression of thyroid-related receptors such as *Thyroid hormone receptor α-1-like, REV-ERBA-α-related receptor, THR α-1, THR β-2, V-ERB-A avian erythroblastic leukemia viral oncogene homolog-like 2 *was also modulated after *BRCA2*-siRNA treatment and the additional phytoestrogen treatments (Figure 3).

**Figure 4:**
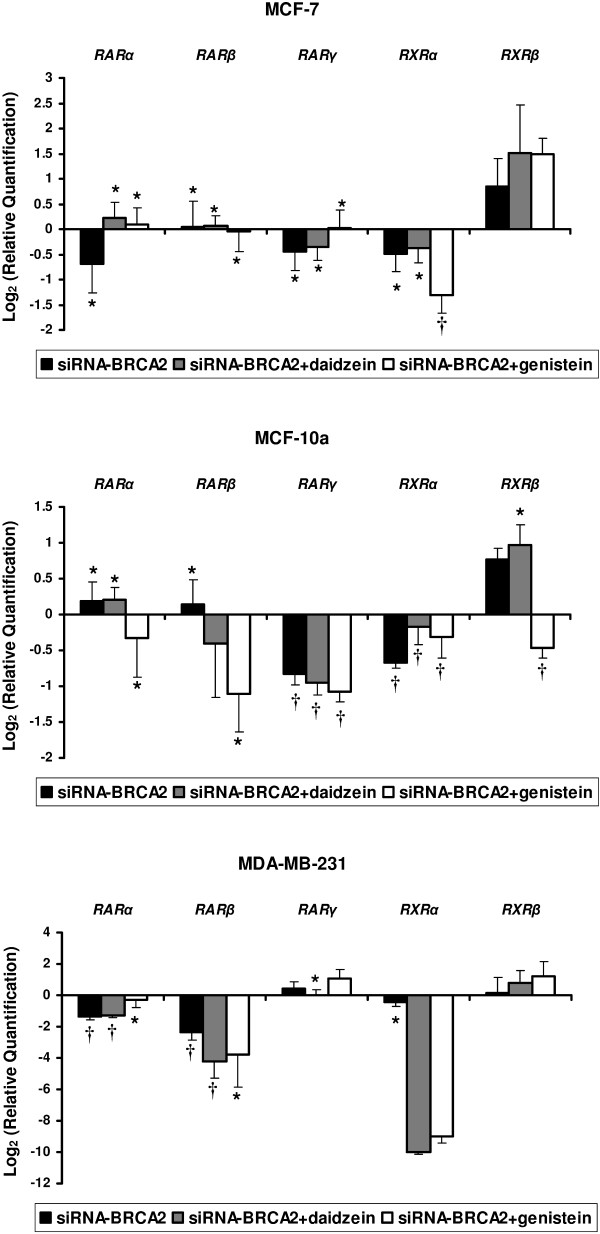
**Modulations in mRNA levels performed by TLDA for retinoic acid receptor-related genes (*RARα*, *RARβ*, *RAR*γ, *RXRα*, and *RXRβ*) occurring after *BRCA2*-siRNA treatment and *BRCA2*-siRNA with isoflavone exposure when compared to control cells corresponding to value 0, in MCF-7, MCF-10a, and MDA-MB-231 cell lines**. Values are means ± SD for n = 3 assays. Statistical analysis were carried out by Student *t*-test (†, *P *< 0.01; *, *P *< 0.05).

We also observed modulation in retinoic acid-related receptors such as *RARα*, *RARβ*, *RAR*γ, *RXRα*, and *RXRβ *after the different treatments (Figure 4).

**Figure 5:**
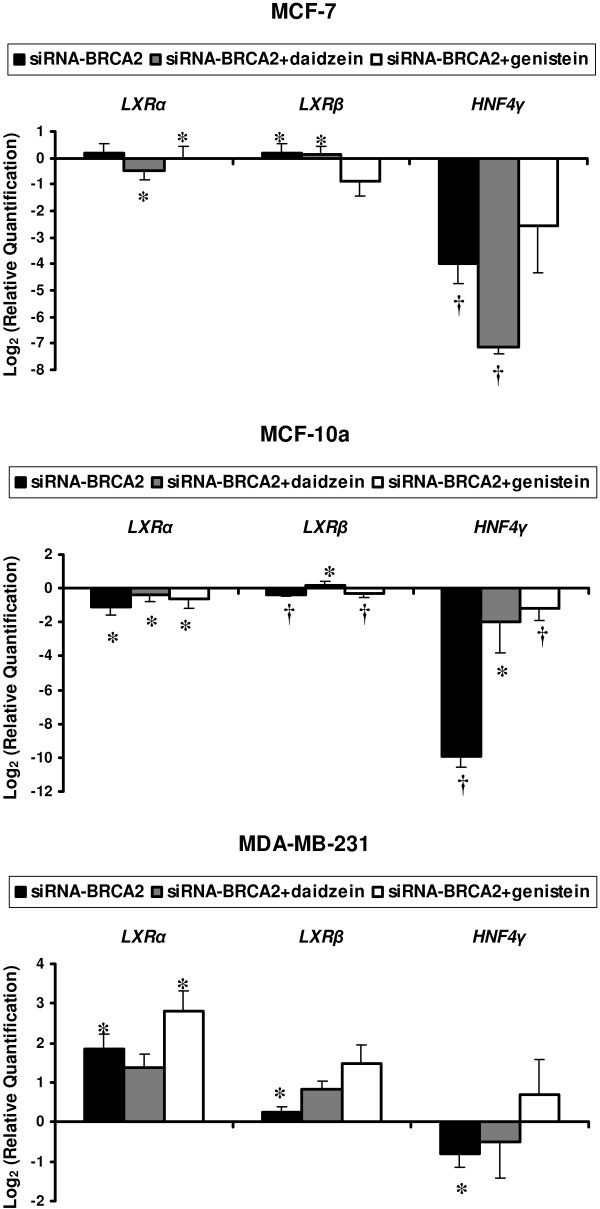
**Modulation in mRNA levels performed by TLDA for liver receptor genes (*LXRα*, *LXRβ *and *HNF4γ*) with differential expression between *BRCA2*-siRNA treatment and *BRCA2*-siRNA with isoflavone exposure when compared to control cells corresponding to value 0, in MCF-7, MCF-10a, and MDA-MB-231 cell lines**. Values are means ± SD for n = 3 assays. Statistical analysis were carried out by Student *t*-test (†, *P *< 0.01; *, *P *< 0.05).

Liver X-related receptors (LXR) α and β expressions were not affected by *BRCA2*-siRNA transfection, except in MDA-MB-231. The hepatocyte nuclear factor (HNF) 4-γ expression was decreased after the both 72 hours treatments (Figure 5).

**Figure 6:**
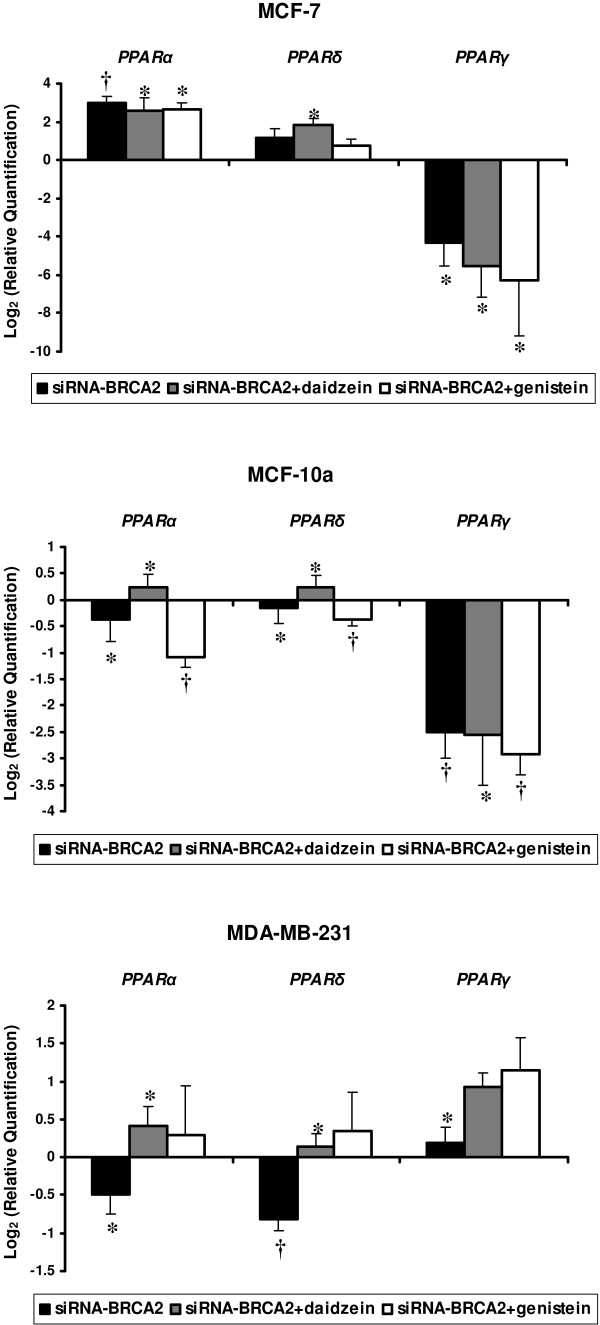
**Modulations in mRNA levels performed by TLDA for peroxisome-related receptor genes (*PPARα*, *PPARδ *and *PPARγ*) with differential expression between *BRCA2*-siRNA treatment and *BRCA2*-siRNA with isoflavone exposure when compared to control cells corresponding to value 0, in MCF-7, MCF-10a, and MDA-MB-231 cell lines**. Values are means ± SD for n = 3 assays. Statistical analysis were carried out by Student *t*-test (†, *P *< 0.01; *, *P *< 0.05).

Peroxisome proliferator-activated receptor (PPARs) α, δ, and γ expressions were also found modulated after the different treatments (Figure 6).

**Figure 7:**
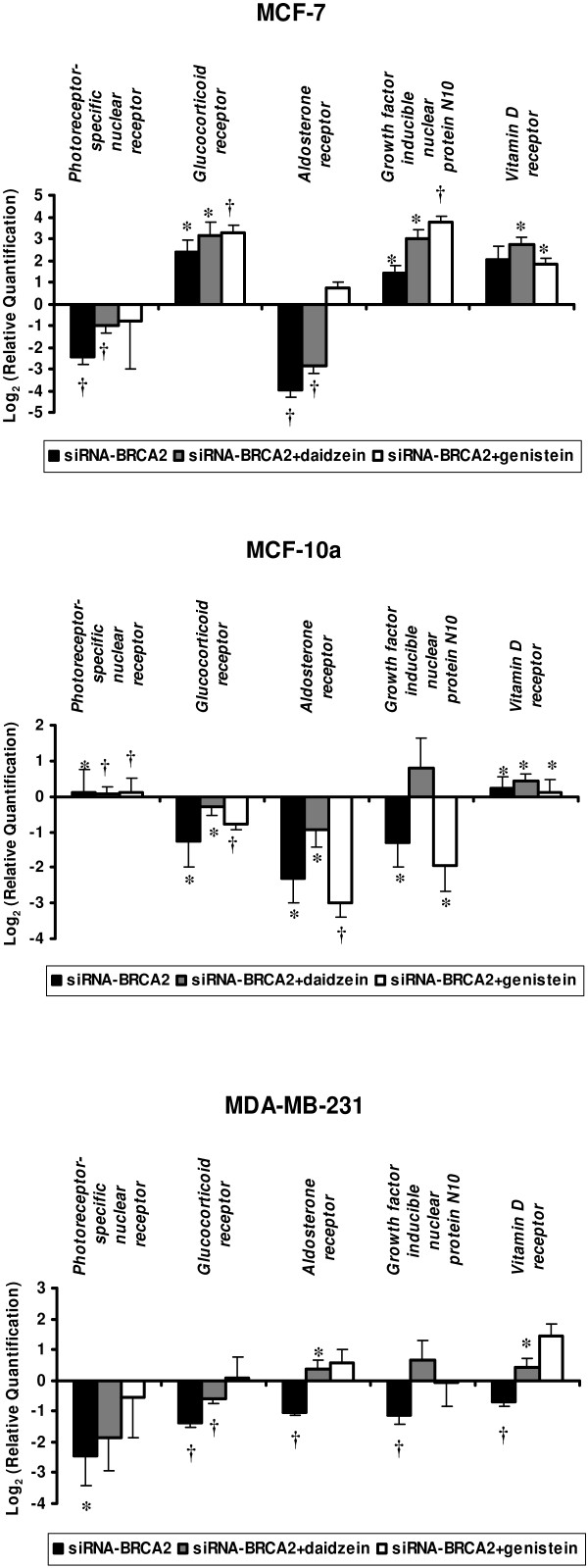
**Modulations in mRNA levels performed by TLDA for hormone receptor genes (*Photoreceptor-specific nuclear receptor, Glucocorticoid receptor, Aldosterone receptor, Growth factor inducible nuclear protein N10 and Vitamin D receptor*) with differential expression between *BRCA2*-siRNA treatment and *BRCA2*-siRNA with isoflavone exposure when compared to control cells corresponding to value 0, in MCF-7, MCF-10a, and MDA-MB-231 cell lines**. Values are means ± SD for n = 3 assays. Statistical analysis were carried out by Student *t*-test (†, *P *< 0.01; *, *P *< 0.05).

Hormone receptors as glucocorticoid, aldosterone, growth factor and vitamin D receptors were studied and showed modulations in their expressions after siRNA-*BRCA2 *transfection and both genistein and daidzein exposures (Figure 7).

## Discussion

Epidemiological evidence suggests that consumption of soy products is inversely correlated with the incidence of certain types of cancers, including breast cancer. Genistein and daidzein, the main soy-associated isoflavones, are considered to be the most important components in soy and have been largely studied in the last years. The cellular and molecular mechanisms involved in these compound actions, however, are not fully understood. Isoflavones are structurally similar to endogenous estrogens of humans and have both estrogenic and antiestrogenic activities. Nuclear receptors are transcriptional factors that play important roles in gene expression regulation and physiological activities. Among these, receptors are sexual hormone receptors, including estrogen receptors (ER) which are critical in breast cancer. This is especially true since most of breast cancers are considered to be estrogen-dependent. Modulation of nuclear receptors seemed to be an important and possible mechanism through which isoflavone such as genistein and daidzein could act on physiological functions.

There is increasing evidence that the silencing of *BRCA2 *gene might be important in the pathogenesis of a significant proportion of sporadic breast cancers where this gene expression is often modulated [15]. To study this, we used RNA interference in breast human cells (MCF-7, MCF-10a, and MDA-MB-231) to down-regulate *BRCA2 *and determine its contribution to nuclear receptor expression at the transcriptional level. Genistein and daidzein 72 hours treatments were added to this transitory loss of function and the expression pattern of 64 nuclear receptor genes were investigated using the TaqMan Low-Density Array (TLDA) technique.

Two subtypes of ER (α and β) were identified and showed distinct tissue-specific distribution patterns. Isoflavones can bind to both ER subtypes due to their structural similarity to human endogenous estrogens. The ratio of *ERα *to *ERβ *is a prognostic marker in breast tumors, in that *ERβ *expression is indicative of more benign tumors, whereas *ERα *indicates malignant, aggressive tumors [16,17]. The three different cell lines used in this study exhibited different ER status: MCF-7 (*ERα*+/*ERβ*+), MDA-MB-231 (*ERα*-/*ERβ*+), and MCF-10a (*ERα*-/*ERβ*-). Under the BRCA2 loss of condition function, we observed in MCF-7, a decreased expression of *ER*α and an increased expression of *ERβ*. Both genistein and daidzein treatments showed the same effects in this cell line. In MDA-MB-231 cell line under siRNA-*BRCA2 *and either siRNA-*BRCA2 *+ genistein or daidzein, we observed a down-regulation of *ERα *and *β*. In MCF-10a, no expression modulation was observed for *ERβ*, and under-expression of *ERα *was observed under siRNA-*BRCA2 *condition. So, in the absence of *BRCA2*, the decreased expression of *BRCA2 *seemed to affect *ER*α mRNA expression in the three cell lines, whereas isoflavone treatments seemed to have no effect on *ER *expression.

The regulation of cell growth and differentiation of normal and malignant cells by retinoids is mediated by the retinoic acid receptors (RARs) and the retinoid × receptors (R×Rs). These are members of the steroid hormone receptor superfamily of transcription factors [18]. Most of known isotypes of RARs and RXRs are expressed in breast cells [19]. Yang & al, 1999 have shown that *RARα *expression is lower in normal breast cells and ER-negative breast cancer cells (MDA MB 231 and MDA MB 435), compared to ER-positive breast cancer cells (MCF7 and T47D) [20]. In our study, *BRCA2 *knockdown is associated with a decreased expression of *RARα *in MCF-7 and MDA-MB-231. After genistein and daidzein 72-h exposures, we observed a restored expression of *RARα *in MCF-7, but this was not the case in MDA-MB-231 cells. This observation may be due to the different ER status exhibited by these two cell lines.

Moreover, loss of *RARβ *has been observed in solid tumor cells, including breast cancer [21]. This finding suggests that the specific loss of *RARβ *expression may be an important event during breast tumorigenesis. This hypothesis is supported by the observation that introduction of *RARβ *gene into breast cancer cell lines restored RA responsiveness to growth inhibition and induction of apoptosis [22]. Other reports have shown that *RARβ *RNA is expressed at low levels in normal and immortal breast cells, but it is absent in ER-positive breast cancer cells [23]. In our study, we observed the absence of *RARβ *described above in MCF-7 ER-positive cells. In MCF-10a, *BRCA2 *knockdown over-expressed *RARβ *when isoflavone treatments decreased its expression. In MDA-MB-231, we observed a general decreased expression of *RARβ *under the different treatments.

It has been reported that *RARγ *is expressed at similar low levels in normal mammary epithelial cells, immortal breast cells, and breast cancer [21]. In MCF-7 and MCF-10a, we observed an under-expression of *RARγ *after both *BRCA2 *knockdown and either genistein or daidzein treatments.

Thyroid hormone receptors (THRs) function as nuclear transcription factors to mediate thyroid hormone actions. They are key regulators of many genes involved in cholesterol and lipid metabolism. They also play important roles in controlling growth, differentiation, development, and carcinogenesis. THR are present in both breast tissue and in breast tumor tissue, although the involvement of these receptors in breast cancer is poorly understood, and their combined effects with estrogens are not well studied [24]. In a recent study, it have been suggested that thyroid hormones and their receptors play a role in breast cancer development and progression in promoting MCF-7 and T47-D cell proliferation, and increasing the effect of 17β-estradiol on cell proliferation [25]. In our study, different profiles were observed between the different cell lines and after the different treatments. *Thyroid hormone receptor α-1-like, REV-ERBA-α-related receptor *and *THR α-1 *were over-expressed in MCF-7 while they were under-expressed in MDA-MB-231 and MCF-10a. Furthermore, no modulation of phytoestrogen treatments was observed on *BRCA2 *knockdown effects.

The peroxisome proliferator activated receptors (PPARs) belong to the nuclear hormone recptor family and play an important regulatory role in lipid metabolism and adipogenesis [26]. Three PPAR genes have been identified in mammalian species: *PPARα*, *PPARβ/δ*, and *PPARγ *[27]. Results of *in vitro *studies demonstrate that the soy isoflavones, particularly genistein and daidzein, were able to activate both PPARα- and PPARγ-mediated gene expressions [28]. Furthermore, genistein has been identified as a ligand of the PPARγ receptor [29]. On recent studies, activation of PPARs has been identified as an approach to induce differentiation and inhibit proliferation of cancer lines. In *ERα*-cell lines MDA-MB-231 and MCF-10a, we observed an under-expression of *PPARα *and *PPARβ/δ *after *BRCA2 *knockdown. It is interesting to note that the addition of genistein or daidzein treatment restored *PPARα *and *PPARβ/δ *expressions in MDA-MB-231. Daidzein treatment restored their expression in MCF-10a cells whereas genistein did not. In MCF-7, we observed a global over-expression of *PPARα *and *PPARβ/δ *and an under-expression of *PPARγ *as observed in MCF-10a. In MDA-MB-231, *PPARγ *were slightly over-expressed after *BRCA2 *knockdown. This increased after genistein or daidzein treatments. These observations suggested that isoflavone may modulate PPARs expression via ER-independent mechanisms.

1α,25-Dihydroxyvitamin D3, the biologically active form of vitamin D that interacts with the vitamin D receptor (VDR), is a coordinate regulator of proliferation, differentiation, and survival of breast cancer cells [30]. On the basis of these findings, there has been considerable interest in the therapeutic use of VDR agonists in the treatment of breast cancer. Furthermore, studies with mice lacking VDRs have established that vitamin D participates in negative growth control of the normal mammary gland and that the disruption of VDR signaling is associated with accelerated mammary tumor development [31]. In MCF-7 and MCF-10a cells, we observed an over-expression of *VDR *after *BRCA2 *knockdown and genistein and daidzein treatments. In MDA-MB-231, we observed a decreased expression of *VDR *after *BRCA2 *knockdown which was reversed after isoflavone treatments.

In conclusion, generating the transitory knockdown of *BRCA2 *oncosuppressor, we observed in three cell lines different modulations in several nuclear receptor genes like *ER*, *RAR *and *RXR*, as well as *PPAR*s, and *VDR*. Additional treatments of genistein and daidzein, the main soy isoflavones, showed the similar or the opposite nuclear receptor gene modulation profiles that we observed under *BRCA2 *knockdown in a cell line independent-manner. These results suggested that many of the modulations observed may implicate, either directly or indirectly, the oncosuppressor *BRCA2 *and an ER-independent cross-modulatory action of genistein and daidzein for several different receptors.

## Abbreviations

ER: estrogen receptor; TLDA: TaqMan Low-Density Array; VDR: vitamin D receptor; PPARs: peroxisome proliferator activated receptors; THRs: Thyroid hormone receptors; LXR: Liver X-related receptors; RAR: retinoic acid-related receptors.

## Competing interests

The authors declare that they have no competing interests.

## Authors' contributions

SS carried out the molecular genetic studies and drafted the manuscript. HS carried out the TLDA assays. NR participated in the TLDA analysis. LF and YJB participated in the design of the study. DBG conceived of the study, and participated in its design and coordination. All authors read and approved the final manuscript.
